# Huang Lian Jie Du Decoction enhances the anti-tumor efficacy of immune checkpoint inhibitors by activating TLR7/8 signalling in melanoma

**DOI:** 10.1186/s12906-024-04444-y

**Published:** 2024-04-11

**Authors:** Suqing Liu, Yaohua Zhang, Xiaohua Zhu, Shan He, Xiao Liu, Xiang Lv, Fuguo Zuo, Jinfeng Wu

**Affiliations:** 1 Department of Dermatology, Huashan Hospital, Fudan University, Shanghai, 200040 China; 2 Worldwide Medical Center, Huashan Hospital, Fudan University, Shanghai, 200040 China; 3 Department of Oncology, Shanghai Municipal Hospital of Traditional Chinese Medicine, Shanghai University of Traditional Chinese Medicine, Shanghai, 200071 China; 4 Department of Dermatology, Shanghai East Hospital, School of Medicine, Tongji University, Shanghai, 200092 China

**Keywords:** Huang Lian Jie Du Decoction, Melanoma, Immunotherapy, Immune checkpoint inhibitors, TLR7/8, IFN-Is

## Abstract

**Background:**

The clinical application of immune checkpoint inhibitors (ICIs) is limited by their drug resistance, necessitating the development of ICI sensitizers to improve cancer immunotherapy outcomes. Huang Lian Jie Du Decoction (HLJD, Oren-gedoku-to in Japanese, Hwangryunhaedok-tang in Korean), a famous traditional Chinese medicinal prescription, has exhibited potential in the field of cancer treatment. This study aims to investigate the impact of HLJD on the efficacy of ICIs in melanoma and elucidate the underlying mechanisms.

**Methods:**

The potential synergistic effects of HLJD and ICIs were investigated on the tumor-bearing mice model of B16F10 melanoma, and the tumor infiltration of immune cells was tested by flow cytometry. The differential gene expression in tumors between HLJD and ICIs group and ICIs alone group were analyzed by RNA-seq. The effects of HLJD on oxidative stress, TLR7/8, and type I interferons (IFN-Is) signaling were further validated by immunofluorescence, PCR array, and immunochemistry in tumor tissue.

**Results:**

HLJD enhanced the anti-tumor effect of ICIs, significantly inhibited tumor growth, and prolonged the survival duration in melanoma. HLJD increased the tumor infiltration of anti-tumor immune cells, especially DCs, CD4^+^ T cells and CD8^+^T cells. Mechanically, HLJD activated the oxidative stress and TLR7/8 signaling pathway and IFN-Is-related genes in tumors.

**Conclusions:**

HLJD enhanced the therapeutic benefits of ICIs in melanoma, through increasing reactive oxygen species (ROS), promoting the TLR7/8 pathway, and activating IFN-Is signaling, which in turn activated DCs and T cells.

**Supplementary Information:**

The online version contains supplementary material available at 10.1186/s12906-024-04444-y.

## Background

Melanoma accounts for 75% of skin cancer-related deaths [1]. Over the past three decades, the incidence of melanoma has exhibited an upward trend due to factors such as an aging population, continued use of tanning beds, increased exposure to sunlight, and heightened awareness and detection [2]. While surgical removal often yields success in treating early-stage melanoma, the outlook for patients with advanced melanoma remains bleak [3]. Significantly, the introduction of immune checkpoint inhibitors (ICIs), a recent addition to the category of immunomodulatory drugs, has substantially improved survival rates in cancer patients [4]. Monoclonal antibodies targeting the immune checkpoint molecule CTLA-4 and PD-1/PD- L1 were approved by the Food and Drug Administration (FDA) for the treatment of advanced melanoma [5]. However, with only 50% of melanoma patients exhibiting long-term responses to ICIs [5], there is an urgent demand for innovative approaches to enhance their effectiveness.

The core mechanism of immune checkpoint inhibitors (ICIs) in cancer treatment revolves around reactivating the adaptive immune system, primarily T cells, to eliminate tumors [6]. This reactivation, crucial for initiating, sustaining, and establishing enduring protective memory T-cell responses within adaptive immunity, hinges on the foundational support of the innate immune response [7]. Within the innate immune response, pattern-recognition receptors (PRRs), like Toll-like receptors (TLRs) [8], play a pivotal role by recognizing conserved pathogen-associated molecular patterns (PAMPs), including bacterial cell walls, proteins, lipopolysaccharides (LPS), and viral RNA/DNA [9, 10]. TLR7 and TLR8, are two members of the TLR family expressed on the surface of the endosome, both of which recognize homologous ligands and trigger the production of proinflammatory cytokines and type I interferons (IFN-Is) in immune cells [11]. Notably, TLR7/8 agonists have found utility as adjuvants in cancer immunotherapy due to their capacity to induce the production of IFN-Is, proinflammatory cytokines, chemokines, and the upregulation of costimulatory molecules through the MyD88 pathway [12]. Consequently, when exposed to these cytokines, dendritic cells and other antigen-presenting cells (APCs) acquire enhanced costimulatory and antigen-presenting capabilities, culminating in the activation of adaptive immune responses [13].

Traditional Chinese Medicine (TCM) has been shown to have efficacy in improving life quality and prolonging the survival time in cancer patients [14, 15], and enhancing the response of radiotherapy, chemotherapy, targeted therapy and photodynamic therapy to cancer [16–20]. Huang Lian Jie Du Decoction (HLJD, Oren-gedoku-to in Japanese, Hwangryunhaedok-tang in Korean), a classical TCM formula with anti-inflammatory effects, has been widely used as combination therapy for cancer [21, 22], arthritis [23], cerebral ischemia/reperfusion [24, 25], type II diabetes [26, 27], and atopic dermatitis [28] in China [29]. It is comprised of *Scutellariae Radix, Coptidis Rhizoma, Phellodendri Chinensis Cortex* and *Gardeniae Fructus*. In this study, we aim to investigate the impact of HLJD on the efficacy of ICIs in melanoma and elucidate the underlying mechanisms based on TLR7/8 signaling.

## Methods

### UHPLC-Q/Preparation of decoction and chromatographic analysis

A total of four herbs in HLJD including *Rhizoma coptidis*, *Radix scutellariae*, *Cortex phellodendri* and *Fructus gardenia* were obtained from Shanghai Hongqiao Chinese Medicine Tablet Co. Ltd. (Shanghai, China), and were further authenticated by the corresponding author Jinfeng Wu as herbs originating form *Scutellaria baicalensis Georgi*, *Coptis chinensis Franch*, *Phellodendron chinense Schneid.*, *Gardenia jasminoides Ellis*, respectively. A voucher specimen for all herbs (HLJD-20,220,905) has been deposited at Specimen Museum of Huashan Hospital, Fudan University.

Epiberberine, berberine, palmatine, coptisine, phellodendrine chloride, baicalin, and gardenoside (1–2 mg each), were dissolved in methanol to obtain stock solutions of each reference substance. These solutions were then mixed into a reference solution at a concentration of 1 µg/mL. *Coptidis Rhizoma* (54 g), *Scutellariae Radix* (36 g), *Phellodendri Chinensis Cortex* (36 g) *and Gardeniae Fructus* (54 g), respectively, add 7 times the amount of water to soak for 30 min, heat and reflux for 60 min, and extract twice. The extract was combined and concentrated under reduced pressure to obtain 38.466 g of extract, and the extract rate was 21.37%. We then took 0.522 g of extract, added water to prepare 40 mL solution, centrifuged at 12,000 rpm at 4 °C for 15 min, transferred the supernatant (100 µL) to the liquid phase vial, and loaded the sample for test. Instrument and chromatographic conditions were the same as described in our previous article [30].

### Reagents

HLJD decoction was prepared as above and stored at − 20 °C. The following flow cytometric antibodies were obtained from Biolegend (San Diego, CA, USA): anti-mouse CD45-APC/Cy7 (Cat#157,617), anti-mouse F4/80-PE (Cat#123,110), anti-mouse CD11b-FITC (Cat#101,206), anti-mouse CD11c-PE/Cy7 (Cat#117,318), anti-mouse CD8a-FITC (Cat#155,004), anti-mouse CD4 PerCP/Cy5.5 (Cat#100,434) and anti-mouse CD3ε-PE/Cy7 (Cat#155,706). Fixable Viability Stain (FVS) 700 was purchased from BD Bioscience (San Jose, CA, USA, Cat#564,997). Primary antibody against mouse TLR7 was purchased from Proteintech (Wuhan, China), and secondary antibodies and DAPI were obtained from Servicebio (Wuhan, China). ROS staining solution (Cat#D7008) was purchased from SIGMA (St. Louis, MO, USA). Mouse anti-PD-1 antibody and anti-CTLA-4 antibody were purchased from Bioxcell (Lebanon, NH, USA).

### Cell line and animals

B16F10 cell line was purchased from the Cell Bank of the China Science Academy (Shanghai, China) and cultured in RPMI 1640 media supplemented with 10% fetal bovine serum (FBS, ScienCell, Carsbad, CA, USA, Cat#0500) at 37 °C in a humidified 5% CO_2_ atmosphere. Six-week-old female C57BL/6 J mice from Zhejiang Vital River Laboratory Animal Technology Co. (Zhejiang, China), were housed in the animal room of Fudan University in a pathogen-free environment. Mice were raised at a constant temperature (25 ± 2 °C) and a proper humidity (60% ± 2%), and were allowed free access to food and water. The light/dark cycle was 12 h light/12 h dark. Fudan University’s animal ethics committee reviewed and approved all animal experiments (202,012,035 S) on 03-12-2020.

### Animal experiments

The therapeutic effects of saline, HLJD, ICIs (anti-PD-1 + anti-CTLA-4), and the combination of HLJD and ICIs were examined on C57BL/6 mice bearing B16F10 tumor. B16F10 cells were suspended in RPMI1640 culture medium without FBS. Suspension of 2.0 × 10^5^ cells in 100 µL medium was injected subcutaneously in the right flank of C57BL/6 J mice. When the tumor volume reached about 40–80 mm^3^, the mice were assigned to 4 groups (6 mice per group) at random: control group (saline, intragastrically (i.g.), every day), HLJD group (i.g., 13.5 g/kg every day), ICIs group (anti-PD-1: intraperitoneally (i.p.), 10 mg/kg; anti-CTLA-4: i.p., 5 mg/kg every three days for three times), and combination of HLJD (i.g., 13.5 g/kg every day) and ICIs (anti-PD-1: i.p., 10 mg/kg; anti-CTLA-4: i.p., 5 mg/kg every three days for three times). The treatment was initiated on day 9. The HLJD dosage for mice was derived from the established safe human dosage. No adverse effects were observed in the mice throughout the experiment. Tumor size was measured every two days with a digital caliper and was calculated as ab^2^/2 (a = the longest diameter, b = the shortest diameter). Tumor volumes were measured once every 2 days and the results were presented with tumor growth curves plotted at day 17. Survival curves were generated for the time until tumors reached the study endpoint (the mouse reached natural death, or the tumor reached the volume of 2000 mm^3^). Mice were euthanized at the experiment endpoint. The mice were anesthetized using an intraperitoneal injection of a mixture of ketamine (100 mg/kg) and xylazine (10 mg/kg). Once the mice were confirmed to be deeply anesthetized by verifying the absence of pedal withdrawal reflex, they were euthanized using cervical dislocation. The methods of anesthesia and euthanasia used in this study adhere to the guidelines set forth in the American Veterinary Medical Association (AVMA) Guidelines for the Euthanasia of Animals, 2020 edition.

### Flow Cytometry Assay

To analyze tumor-infiltrating immune cells, the tumors were first excised and minced into small pieces. The tissue fragments were then filtered through a 40 μm cell strainer to remove larger tissue pieces, followed by two washes to obtain a single-cell suspension. Subsequently, the cells were treated with 1x red blood cell lysis buffer to eliminate erythrocyte contamination. Finally, following washing and cell counting, the cells were processed through the appropriate FACS staining protocol as previously described [31]. Dendritic cells (DCs) were then stained with anti-mouse antibodies against CD45, F4/80, CD11b and CD11c, and T cells were labelled with antibodies against CD45, CD3, CD4, and CD8, according to the manufacturer’s protocols. We performed flow cytometry with the BD LSRFortessa and analyzed the data with FlowJo (Ashland, OR, USA).

### Immunohistochemistry and immunofluorescence

Immunohistochemistry was performed as described in our previous article [32]. Briefly, after being fixed with 4% formaldehyde, the tumor tissue was paraffin-embedded and sliced into 8–10 μm thick sections and incubated with TLR7 primary antibodies. Immunofluorescence was used to measure ROS levels in the tumor tissue. Fresh-frozen tumor tissue was serially sectioned at 8–10 μm thickness and incubated in ROS staining solution at 37 °C for 30 min in the dark. Then stained with DAPI solution at room temperature for 10 min and kept in a dark place. After washing three times with PBS in a Rocker device, the images were acquired by Fluorescent Microscopy (NIKON Eclipse Ci, Japan).

### RNA sequencing (RNA-Seq) analysis

The miRNeasy Micro Kit (Qiagen, Hilden, Germany) was used to isolate the total RNA of tumor tissue. RNA concentration and purity were determined with the Bioanalyzer 4,200 (Agilent, Santa Clara, CA, USA). The RNA-seq analysis was conducted following the detailed methodology described in our previous study [32].

### qRT-PCR array

Based on the manufacturer’s protocol (Wcgene Biotech, Shanghai, China), gene expression profiles were analyzed using mouse Oxidative Stress PCR Array plate and mouse type I IFN PCR Array plate. Data were analyzed using Wcgene Biotech software. It was considered that genes with fold changes higher than 2 or lower than − 2 were biologically significant.

### ELISA

The level of IFN-α in the tumor tissue was measured by Mouse IFN-α ELISA kit (QuantiCyto, EMC035a) following the manufacturer’s instructions.

### Statistical analysis

All statistical analyses were performed using GraphPad Prism 8 software (GraphPad Software, Inc., San Diego, CA, USA). The data were expressed as the median values ± standard deviation (SD). The statistical significance of the differences between the two groups was analyzed using the student’s t-test, and comparisons among multiple groups by one-way analysis of variance followed by Tukey’s post hoc test. Tumor volume differences were analyzed using two-way ANOVA and Tukey’s test for multiple comparisons. Survival data were analyzed using Kaplan-Meier survival analysis. *P* value less than 0.05 were regarded as a statistically significant difference. (*p < 0.05, **p < 0.01, ***p < 0.001, ****p < 0.0001).

## Results

### The active ingredients of HLJD

**Fig. 1:**
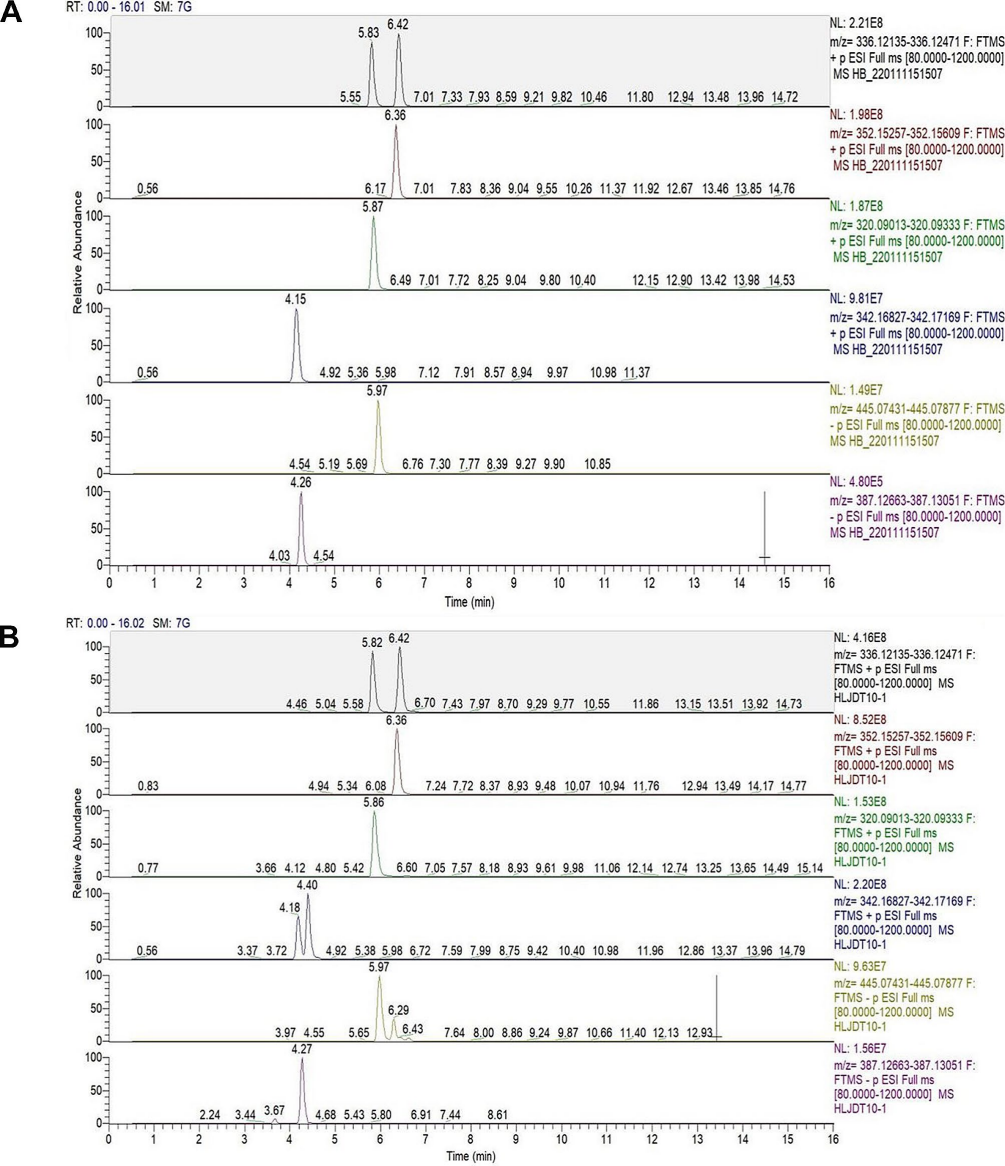
Representative full-scan chromatograms of (**A**) Standard solutions; (**B**) Sample solution: Epiberberine (5.82 min), berberine (6.42 min), palmatine (6.36 min), coptisine (5.86 min), Phellodendrine chloride (4.18 min), baicalin (5.97 min), gardenoside (4.27 min)

To analyze the pharmacological basis of the drug substance, chromatograms of mixed standards and HLJD were compared to properly characterize the components of the HLJD decoction (Fig. 1). We found that epiberberine, berberine, coptisine, palmatine, phellodendrine chloride, baicalin and gardenoside in HLJD were approximately detected to be 5332.42, 3711.690, 13652.51, 1069.15, 2243.74, 29163.21, 21334.80 µg/g, respectively. These results indicated that epiberberine, berberine, coptisine, palmatine, phellodendrine chloride, baicalin and gardenoside might be the active ingredients of HLJD in treating disease.

### HLJD improved ICI’s inhibition of tumor growth and increased survival in melanoma-bearing mice

**Fig. 2:**
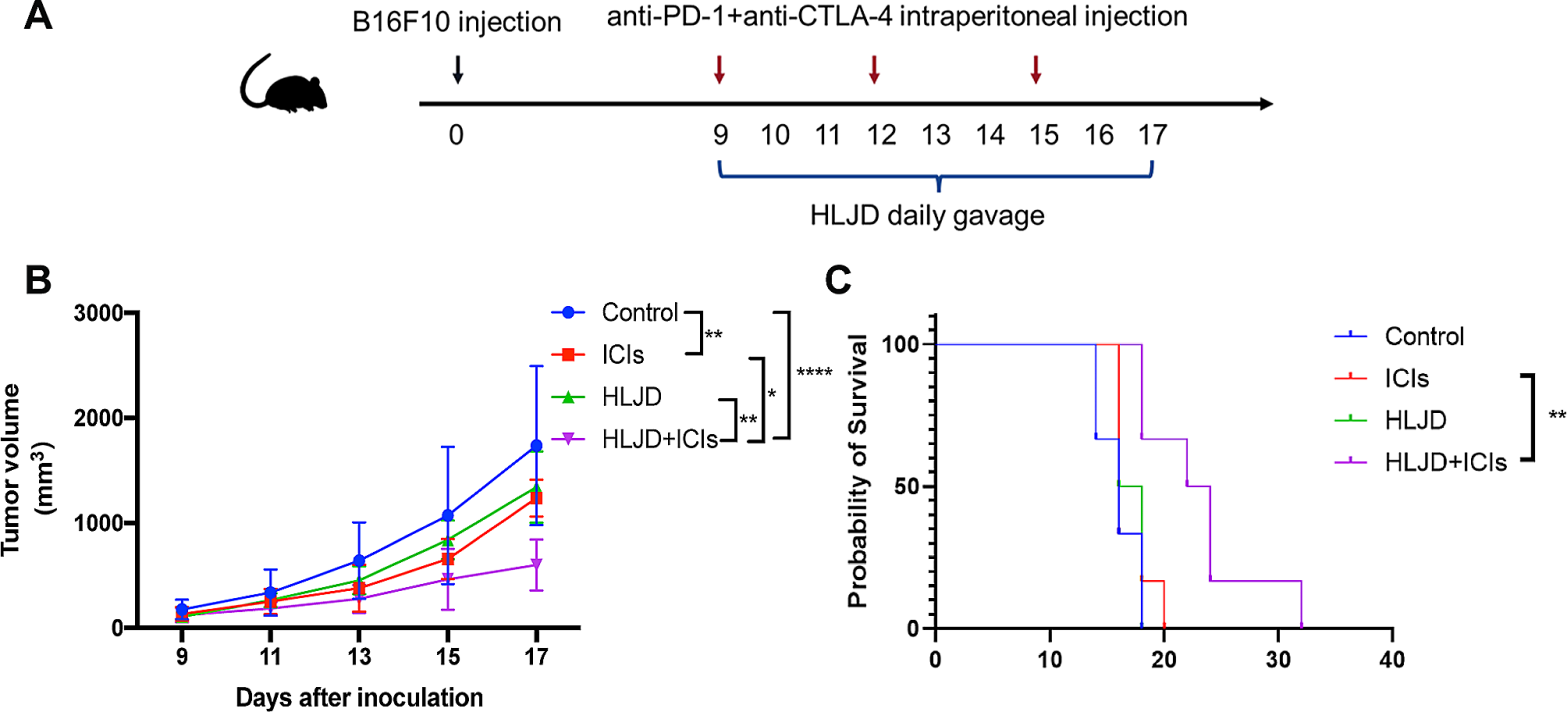
Effects of HLJD for the treatment of B16F10 growth in vivo. (**A**) Schematic illustration of the experiment design. (**B**) Tumor growth curves on B16F10-tumor-bearing mice with different treatments as indicated. (**C**) Survival curves of mice in each group. (*n* = 6 mice/group). Differences in tumor volume were evaluated using two-way ANOVA with post hoc Tukey’s multiple comparisons test. Survival outcomes were assessed by Kaplan-Meier analysis. (*p < 0.05, **p < 0.01, ****p < 0.0001). HLJD = Huang Lian Jie Du Decoction, ICIs = anti-PD-1 + anti-CTLA-4

To evaluate the effects of HLJD in enhancing the response of ICIs, we established a tumor-bearing animal model of B16F10 melanoma and initiated intervention with HLJD and ICIs on the 9th day following B16F10 cell inoculation. (Fig. 2A and Supplemental Table 1). We found that the HLJD + ICIs group markedly suppressed melanoma growth (Fig. 2B), and had a higher survival (*P* < 0.01, Fig. 2C), as compared to ICIs alone. There were no significant differences in body weight change between the groups (data not shown). The above data demonstrated that HLJD improved ICI’s inhibition of tumor growth and increased survival in melanoma-bearing mice.

### HLJD increased tumor infiltration of DCs, CD4^+^T and CD8^+^T cells in tumor

**Fig. 3:**
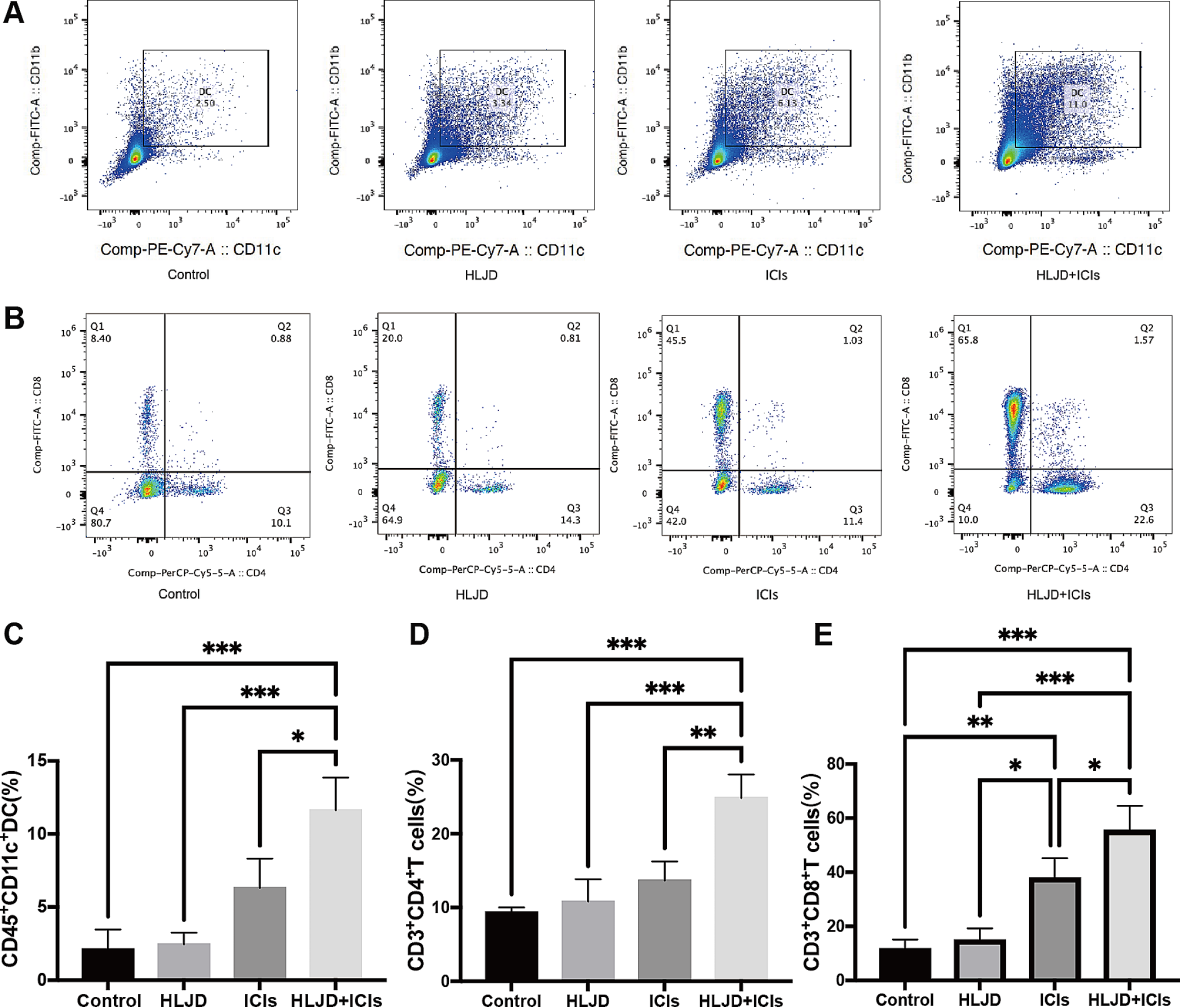
Analysis of tumor immune cell infiltration. (**A** and **B**) Representative flow cytometric plot of DCs, CD4^+^T cells and CD8^+^T cells in tumors. (**C**-**E**) The percentages of DCs, CD4^+^T cells and CD8^+^T cells in tumors following different treatments. (*n* = 3 mice/group) Statistical differences between treatment groups were determined using one-way ANOVA with subsequent Tukey’s post hoc analysis for multiple comparisons. (*p < 0.05, **p < 0.01, ***p < 0.001). HLJD = Huang Lian Jie Du Decoction, ICIs = anti-PD-1 + anti-CTLA-4

The infiltration of immune cells plays a vital role in ICI response [33]. On the 17th day after tumor inoculation, the infiltration of immune cells in the tumor site was analyzed by flow cytometric (Fig. 3A and B). We found that compared with the treatment by ICIs alone or HLJD alone, HLJD combined with ICIs significantly increased the tumor infiltration of DCs (Fig. 3C), CD4^+^T and CD8^+^T cells (Fig. 3D, E). Tumor infiltration of MDSCs and macrophages was not differentially affected (Supplemental Fig. 1). Altogether, HLJD increased tumor infiltration of DCs, CD4^+^T and CD8^+^T cells in tumor.

### HLJD upregulated TLR7/8 pathways in tumor

**Fig. 4:**
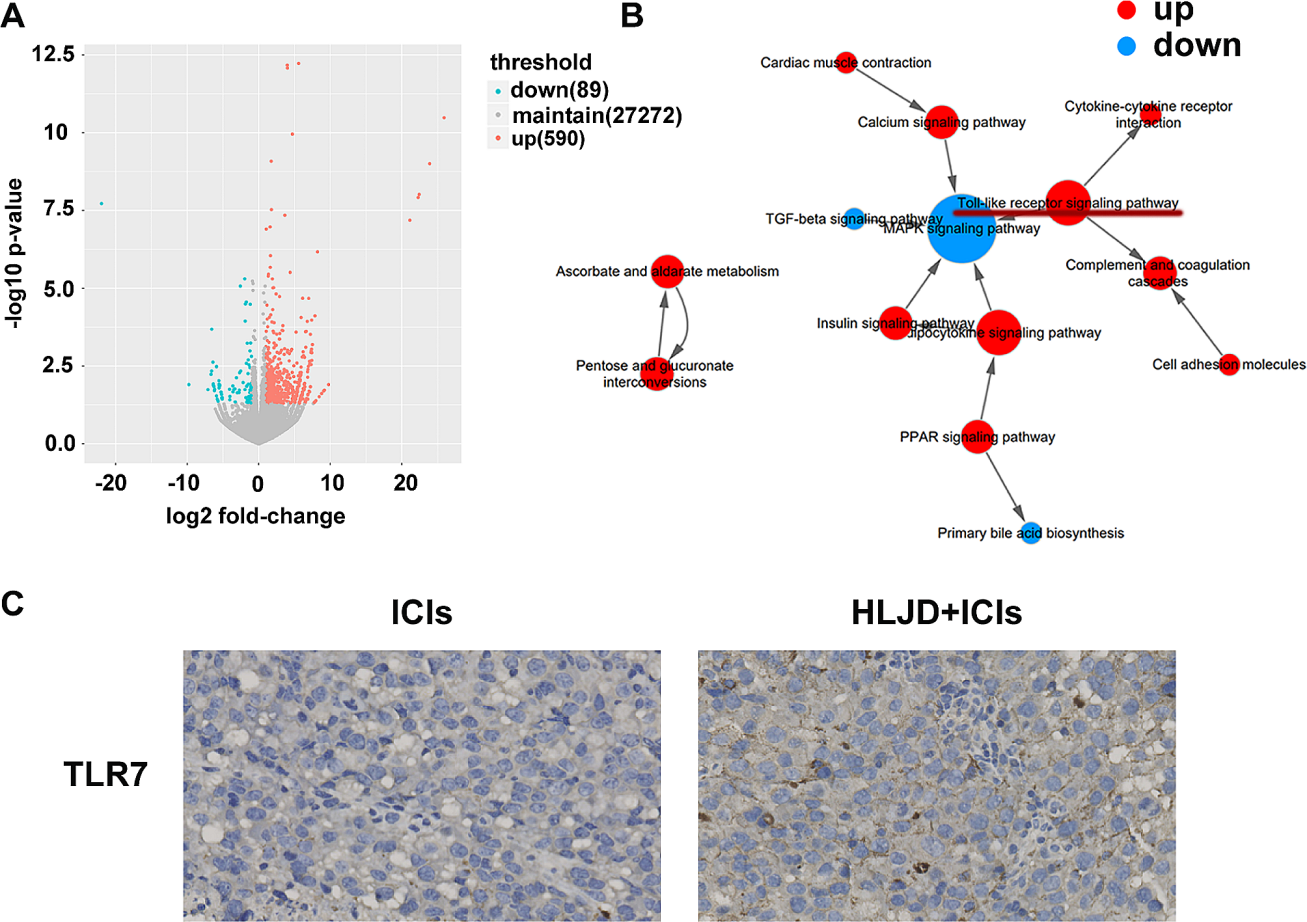
Toll-like receptor signaling pathway is activated after HLJD treatment. (**A**) RNA-seq analysis was performed and the volcano plot was demonstrated. (*n* = 3 mice/group) (**B**) Interaction net of the significant pathways (Path-Net) of differentially signaling pathways. (**C**) Immunohistochemistry was used to analyze the expression levels of TLR7 (400×). HLJD = Huang Lian Jie Du Decoction, ICIs = anti-PD-1 + anti-CTLA-4

To further explore the mechanistic insights into the beneficial effect of HLJD, we conducted RNA-seq analysis using tumor tissue from the HLJD + ICIs group and ICIs alone group. A total of 89 downregulated genes and 590 upregulated genes were found in the HLJD + ICIs group, as compared to the ICIs alone group (Fig. 4A, *n* = 3 mice/group). To further understand the interactions between significant signaling pathways, an interaction net was built using Path-Net analysis. As shown in Fig. 4B, combined HLJD and ICIs treatment increased activation of the TLR signaling pathway, as compared to ICIs alone. The mRNA expression of TLR7 (1.45 fold change) and TLR8 (1.24 fold change) in the HLJD + ICIs treatment group were upregulated, as compared to the ICIs treated alone group. The protein expression of TLR7 was confirmed by immunohistochemistry analysis (Fig. 4C), which is in line with the change in mRNA expression.

### HLJD activated type I IFN signaling in tumor

**Fig. 5:**
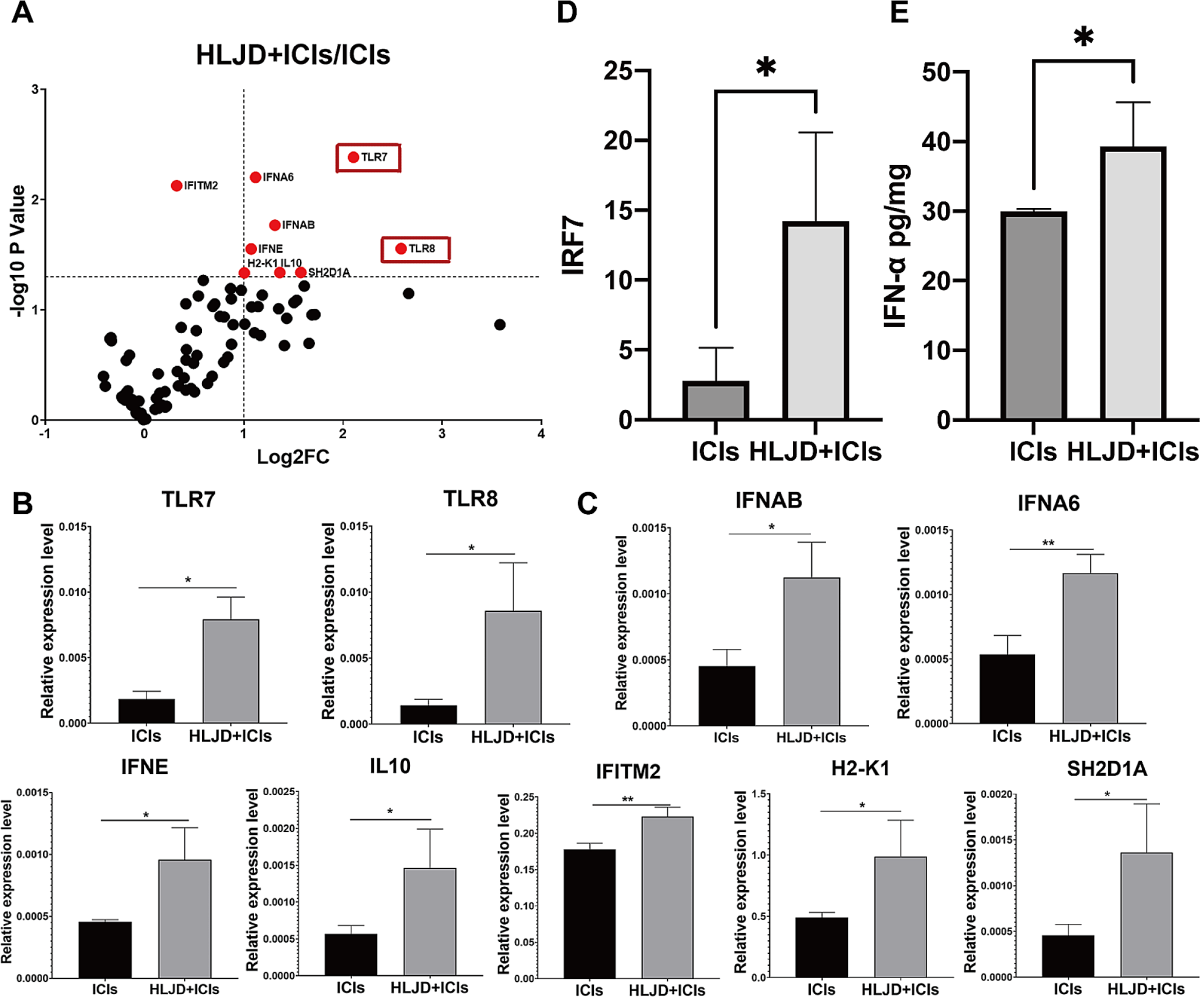
HLJD treatment upregulated Type I IFN signaling. (**A**, **B** and **C**) Volcano Plot graph and Bar graphs of PCR-array of TLR7/8 and Type I IFN signaling axis. (**D**) The expression of IRF7 was determined by qRT-PCR. (**E**) IFN-α quantification by ELISA in tumor tissue. Statistical differences were determined using the student’s t-test. (*p < 0.05, **p < 0.01) HLJD = Huang Lian Jie Du Decoction, ICIs = anti-PD-1 + anti-CTLA-4

TLR7 induces IFN-Is and pro-inflammatory cytokine production [34, 35]. We next investigated the expression of Type I IFN signalling-associated genes using PCR array. Results found that the mRNA levels of many IFN-Is-related genes were upregulated, including TLR7, TLR8, IFNAB, IFNA6, IFNE, IL10, IFITM2, H2-K1, and SH2D1A, after HLJD and ICIs treatment (Fig. 5A, B, and C). We further detected the transcription level of IRF7, which is one of the major regulators in the IFN-I signaling, and found it was also increased in the HLJD and ICIs treated group (Fig. 5D). IFN-α is one of the major products of IFN-I signaling, which can be regulated by IRF7. In addition, the level of IFN-α in the HLJD and ICIs treated group was much higher than that in the ICIs alone group (Fig. 5E). These findings suggested that HLJD activated TLR7/8 and Type I IFN signaling in tumors.

### HLJD increased the oxidative stress in the tumor

**Fig. 6:**
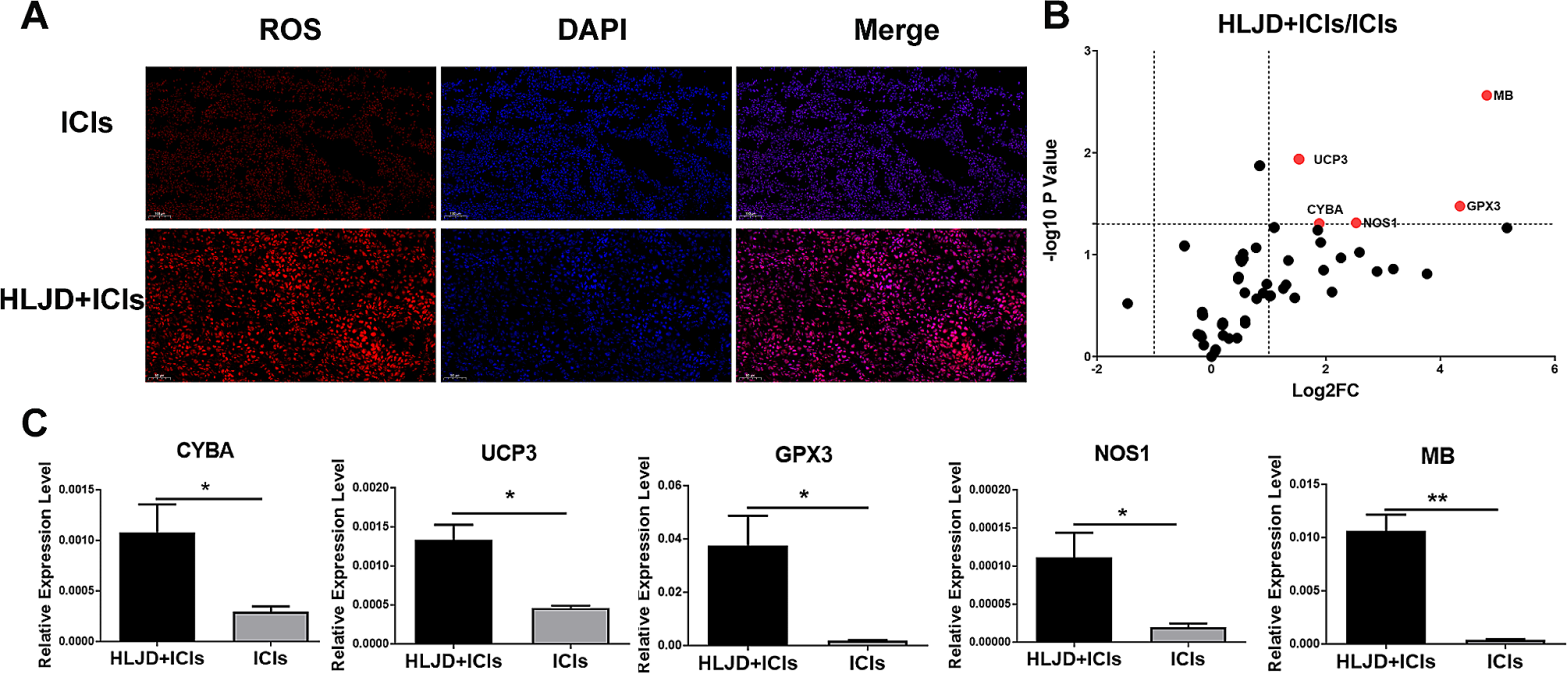
HLJD increased oxidative stress in tumor tissue. (**A**) Representative immunofluorescence images of ROS in tumor tissue (200×). (**B** and **C**) Volcano Plot graph and Bar graphs of PCR-array of oxidative stress-related genes. Statistical differences were determined using the student’s t-test. (*p < 0.05, **p < 0.01) HLJD = Huang Lian Jie Du Decoction, ICIs = anti-PD-1 + anti-CTLA-4

Activation of TLR pathways and oxidative stress can mutually promote each other [36], and IFN-I enhances the production of ROS [37]. To investigate how the HLJD contributes to the activation of TLR and IFN-I signaling pathways, we tested the ROS in tumors using immunofluorescence. As shown in Fig. 6A, ROS was markedly increased in the HLJD + ICI group, as compared to the ICI alone group. We also detected the mRNA expression levels of oxidative stress-associated genes by PCR array and found upregulation of the expressions of CYBA, GPX3, NOS1, UCP3, MB after combined HLJD and ICI treatment (Fig. 6B, C). These results indicated that HLJD increased the oxidative stress in the tumor.

## Discussion

TCM decoctions have been increasingly recognized for their role as ICI sensitizers in oncology. Notably, our study is the first to report the immunotherapy sensitizing effects of HLJD decoction. Beyond HLJD, other TCM formulations have also shown promise. Gegen Qinlian Decoction (GQD) was found to enhance anti-PD-1 monoclonal antibody therapy by modulating the gut microbiome and certain metabolic pathways, which in turn improved immune responses in colorectal cancer models [38]. Similarly, Dahuang Fuzi Baijiang Decoction (DFBD) showed efficacy in enhancing CD8^+^ T cell infiltration and reducing exhaustion markers like PD-1 in tumor microenvironments, although it did not exhibit a synergistic effect with ICIs [39]. Qingfei Jiedu Decoction (QFJDD) acted on lung cancer cells by downregulating PD-L1 expression and affecting key signaling pathways involved in the PD-1/PD-L1 axis [40]. Finally, YIV-906, a standardized botanical drug inspired by TCM, in combination with anti-PD1, eradicated Hepa 1–6 tumors in mice and was proposed to work by enhancing both adaptive and innate immunity, reducing immune tolerance, and influencing macrophage polarization [41]. These findings suggest that TCM decoctions could be valuable adjuncts to ICI therapy by targeting multiple facets of the immune response in cancer.

HLJD Decoction is a well-known prescription of TCM with the ability to regulate inflammation and oxidative stress [42]. HLJD may block hepatocellular carcinoma progression [22] and thus has potential as a new complementary agent for cancer treatment. In this study, the melanoma tumor-bearing mice treated with HLJD and ICIs resulted in a significant reduction in tumor volume and an increased survival rate compared to mice treated with ICIs alone or HLJD alone, suggesting that HLJD could enhance the therapeutic benefits of ICIs in the treatment of melanoma.

We initially tried a low dose of HLJD (4.5 g/kg), which did not enhance the efficacy of ICIs (data not shown). Then we increased the dose of HLJD to 13.5 g/kg, which reached the expected effect. This data suggests that the administration dose should be optimized to produce an improved therapeutic effect in future studies. As there are seven major components in HLJD, including epiberberine, berberine, palmatine, coptisine, phellodendrine chloride, baicalin, and gardenoside, further study is necessary to identify the bioactive components in HLJD that improve ICI response.

At the end of the experiment, the tumor tissues were removed and isolated for flow cytometry analysis. The tumor infiltrating DCs, CD4^+^T and CD8^+^T cells were substantially increased after HLJD and ICIs co-treatment. However, our current analysis focused solely on quantifying immune cell numbers. Future investigations assessing the subtypes and functions of these immune cells, including in vivo CD8^+^T depletion experiments, would provide a more comprehensive understanding of their roles in tumor suppression. TLR 7/8 agonists have been investigated as potential vaccine adjuvants because they can activate APCs and enhance both cellular and humoral immune responses [43]. Activation of TLR7 triggers a cascade of events through a pathway that relies on MyD88, leading to the secretion of inflammatory cytokines like TNF-α, IFN-α, IFN-β, and IL-12, which enhance T cell immunity [44]. Intratumoral injection of TLR7/8 agonists provides superior antigen presentation and co-stimulatory activity [45]. Imiquimod, a famous TLR7 agonist, is approved for the topical treatment of superficial basal cell carcinoma [46]. When used in combination with chemotherapeutic agents, a phase II clinical trial found imiquimod to be effective in treating treatment-refractory breast cancer chest wall metastases [47]. Many TLR7/8 agonists combined with nanoparticles have been studied in cancer immunotherapy and photothermal therapy [48, 49]. In this study, we found that HLJD significantly upregulated the expression of TLR7 and TLR8 and activated IFN-Is signaling in melanoma tissues, acting in part as a safe and cost-effective TLR agonist. Interferon Regulatory Factors (IRFs) are transcription factors that are important in controlling gene networks for coordinating appropriate and effective immune responses [50]. The IRF gene family consists of 9 members, among them, IRF3 and IRF7 are highly homologous and the major regulators in the IFN-I signaling [51]. We also found the expressions of IRF7 and IFNα were markedly increased in the HLJD and ICIs treated group.

Activation of TLR pathways and oxidative stress can mutually affect each other [36]. Imiquimod, a classic TLR7 agonist induces ROS production, disrupting the balance of mitochondrial dynamics [52]. The ROS may enhance DC-mediated anti-tumor immune responses through the promotion of STING [53]. In this study, we found that HLJD markedly increased the production of ROS in melanoma. Several drugs and phototherapy enhance the antitumor response of ICIs by increasing ROS [54, 55]. Future studies, including in vitro analyses, are essential to ascertain whether ROS scavengers can reverse HLJD-induced ICI sensitization and to delineate the direct molecular effects of HLJD on melanoma cells. It has been reported that berberine, one of the major components in HLJD, could upregulate intracellular ROS level in cancer cells [56], which support that berberine might be one of the potentially bioactive substances in HLJD for sensitizing ICIs. Since HLJD has been used for hundreds of years with good safety, it might be a promising candidate drug as an ICI sensitizer.

## Conclusion

**Fig. 7:**
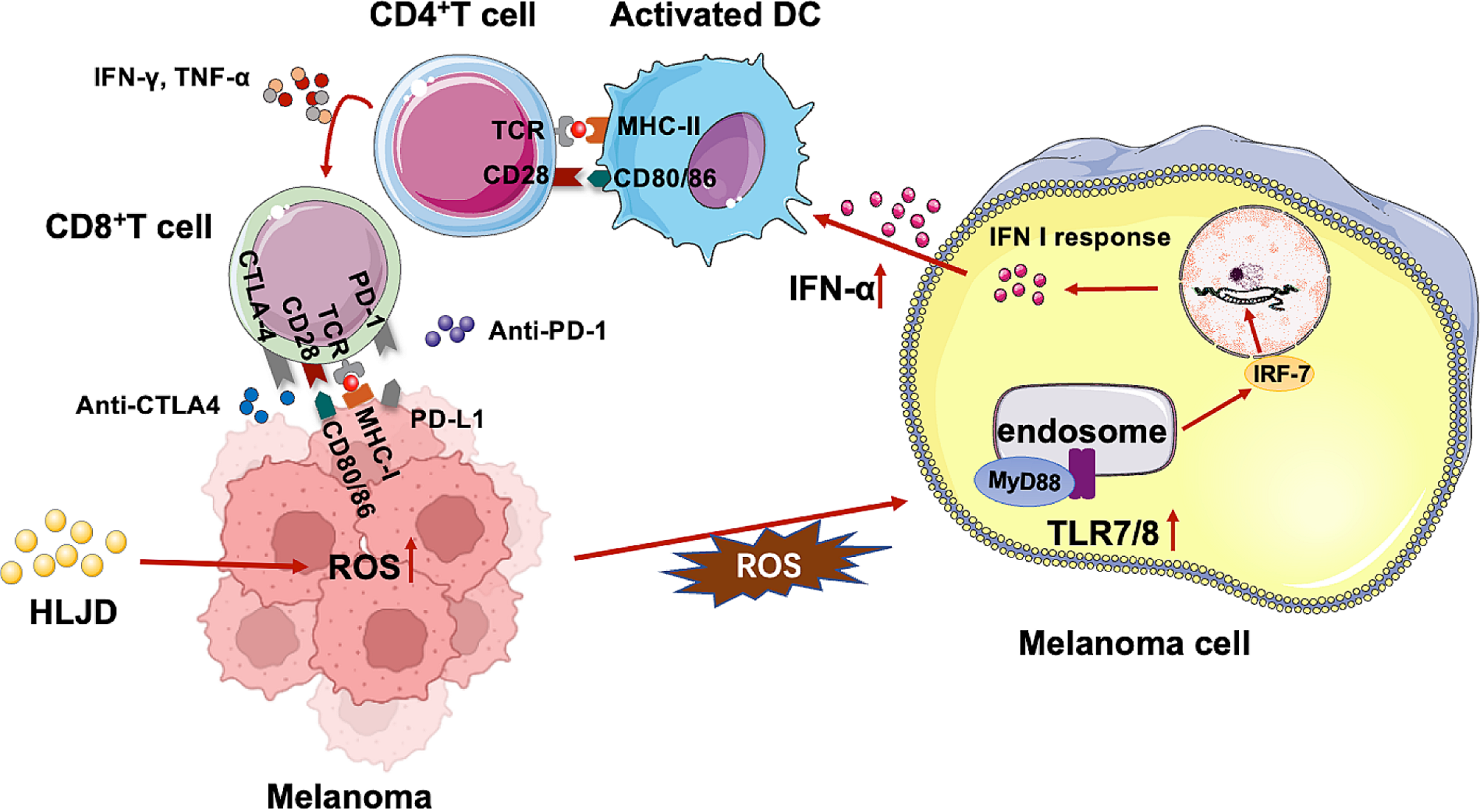
Schematic diagram of mechanisms about enhancement of therapeutic benefits of ICIs in melanoma using HLJD. HLJD = Huang Lian Jie Du Decoction, ICIs = anti-PD-1 + anti-CTLA-4

HLJD enhanced the therapeutic benefits of ICIs in melanoma. Mechanistically, HLJD activated DCs and T cells by increasing ROS, activating the TLR7/8 pathways, and inducing IFN-Is signaling (Fig. 7).

### Electronic supplementary material

Below is the link to the electronic supplementary material.

Supplementary Material 1: Figure S1

Supplementary Material 2: Table S1

## Data Availability

In this study, datasets are available in online repositories. The repository/repositories and accession numbers are listed below: Sequence Read Archive (SRA), PRJNA822503.

## Abbreviations

ICIs: immune checkpoint inhibitors
HLJDD: Huang Lian Jie Du Decoction
IFN-Is: Type I interferons
FDA: Food and Drug Administration
i.g.: intragastrically
i.p.: intraperitoneally
PRRs: Pattern-recognition receptors
PAMPs: Pathogen-associated molecular patterns
LPS: Lipopolysaccharides
TLRs: Toll-like receptors
ssRNA: Single-stranded RNA
APCs: antigen presenting cells
TCM: Traditional Chinese Medicine
ROS: Reactive oxygen species
DCs: Dendritic cells
IRFs: Interferon regulatory factors
