# Supplementary material for: Huang Lian Jie Du Decoction enhances the anti-tumor efficacy of immune checkpoint inhibitors by activating TLR7/8 signalling in melanoma
Source: BMC Complement Med Ther. 2024 Apr 11;24:156. doi: 10.1186/s12906-024-04444-y (PMC11007990; doi:10.1186/s12906-024-04444-y)

**Supplemental Figure 1. Flow cytometry analysis of immune cell populations in tumor tissue.** (A) Gating strategy for CD8^+^ and CD4^+^ T cells. (B) Gating strategy for DCs. (C and D) The percentage of Monocytic-MDSCs (M-MDSC) and Polymorphonuclear-MDSCs (PMN-MDSC) in tumors following different treatments. (E and F) The percentage of M1 macrophages and M2 macrophages in tumors following different treatments. (*p < 0.05) HLJD=Huang Lian Jie Du Decoction, ICIs=anti-PD-1+anti-CTLA-4.


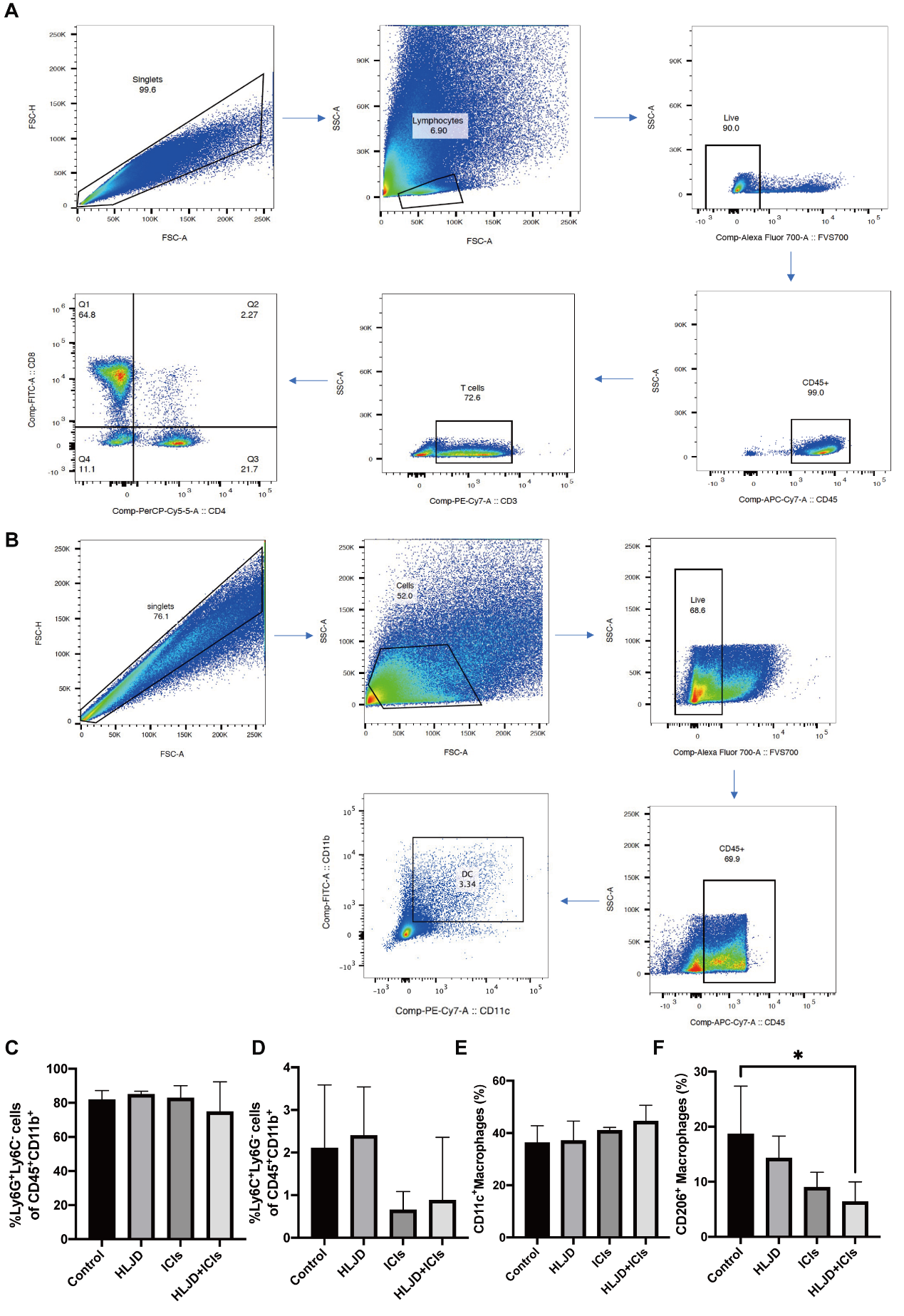

Supplement: Supplementary file 1 — Supplementary Material 1: Figure S1 [file 12906_2024_4444_MOESM1_ESM.docx]
