# Supplementary material for: Huang Lian Jie Du Decoction enhances the anti-tumor efficacy of immune checkpoint inhibitors by activating TLR7/8 signalling in melanoma
Source: BMC Complement Med Ther. 2024 Apr 11;24:156. doi: 10.1186/s12906-024-04444-y (PMC11007990; doi:10.1186/s12906-024-04444-y)
Supplement: Supplementary file 2 — Supplementary Material 2: Table S1 [file 12906_2024_4444_MOESM2_ESM.docx]

| Group | Treatment1 | Dosage | administration | Frequency | Treatment2 | Dosage | administration | Frequency |
| --- | --- | --- | --- | --- | --- | --- | --- | --- |
| Control | saline | 0.3 ml | i.g. | daily | saline | 0.2 ml | i.p. | every three days, 3 times |
| ICIs | saline | 0.3 ml | i.g. | daily | anti-PD-1; anti-CTLA-4 | 10 mg/kg; 5 mg/kg | i.p. | every three days, 3 times |
| HLJD | HLJD Decoction | 0.3 ml (13.5 g/kg) | i.g. | daily | saline | 0.2 ml | i.p. | every three days, 3 times |
| HLJD+ICIs | HLJD Decoction | 0.3 ml (13.5 g/kg) | i.g. | daily | anti-PD-1; anti-CTLA-4 | 10 mg/kg; 5 mg/kg | i.p. | every three days, 3 times |

**Supplemental Table 1. Summary of Animal Experimental Groups and Dosing Regimens.**
